# Fabrication of Structural-Coloured Carbon Fabrics by Thermal Assisted Gravity Sedimentation Method

**DOI:** 10.3390/nano10061133

**Published:** 2020-06-08

**Authors:** Jiali Yu, Cheng Hao Lee, Chi-Wai Kan, Shixin Jin

**Affiliations:** Institute of Textile and Clothing, The Hong Kong Polytechnic University, Hung Hom, Kowloon, Hong Kong 999077, China; scarlett.yu@connect.polyu.hk (J.Y.); chenghao.lee@polyu.edu.hk (C.H.L.); jason.jin@polyu.edu.hk (S.J.)

**Keywords:** structural colour, carbon fabric, photonic crystal, gravity sedimentation

## Abstract

Structural-coloured poly(styrene-methyl methacrylate-acrylic acid) (Poly(St-MMA-AA)) deposited carbon fabrics (Poly(St-MMA-AA)/PCFs) with fascinating colours (salmon, chartreuse, springgreen, skyblue, mediumpurple) changing with the (Poly(St-MMA-AA) nanoparticle sizes can be facilely fabricated by the thermal-assisted gravity sedimentation method that facilitates the self-assembly of Poly(St-MMA-AA) colloidal nanoparticles to generate photonic crystals. The particle sizes of Poly(St-MMA-AA) copolymer with core/shell structure varying from 308.3 nm to 213.1 nm were controlled by adjusting the amount of emulsifier during emulsion polymerisation. The presence of the intrinsic chemical information of Poly(St-MMA-AA) copolymer has been ascertained by Raman and Fourier Transform Infrared (FT-IR) Spectroscopy analysis. Colour variation of the as-prepared structural-coloured carbon fabrics (Poly(St-MMA-AA)/PCFs) before and after dipping treatment were captured while using an optical microscope. The structural colours of Poly(St-MMA-AA)/PCFs were assessed by calculating the diffraction bandgap according to Bragg’s and Snell’s laws. The Poly(St-MMA-AA) photonic crystal films altered the electrical properties of carbon fabrics with the resistivity growing by five orders of magnitude. The differential electrical resistivity between Poly(St-MMA-AA)/PCFs and wet Poly(St-MMA-AA)/PCFs combined with the corresponding tunable colours can be potentially applied in several promising areas, such as smart displays, especially signal warning displays for traffic safety.

## 1. Introduction

Photonic crystal, an artificial crystal with highly periodic structures that are allowed to manipulate the propagation of light, has emerged as one of the promising optical materials for developing inherent structural colours, the studies of such materials possessing intrinsic structural colours by adjusting the photonic crystal bandgap into visible spectrum range has been conducted over the past few decades [1,2,3]. The bandgap derived from highly ordered periodic structures is responsible for uniformly reflected colours in the visible spectrum. Accordingly, a variety of techniques, like photolithography [4], screen printing [5], atomisation deposition [6], inkjet printing [7,8], spray coating [9,10], gravity sedimentation [11,12], and electrophoretic deposition [13,14], have been proposed for the adhibition by applying photonic crystals onto substrates in several applications, including paints and inks [5], smart windows [3], energy-saving devices [15], night-time traffic safety and advertisement display [16], anti-counterfeiting security [4,17,18], eyeglasses and photographic lenses [19], and information storage [20]. To realise such promising industrial applications, the studies on the large-area scalable fabrication technique of photonic crystals have been conducted, including melt-shear organization method [21] and oscillatory shear technique [22]. Zhao et al. [23] achieved large-scale assembly of polymer opals via bending-induced oscillatory shear technique that was equipped with a roll-to-roll setup. Baumberg et al. [24] presented the fabrication of polymer opals possessing tuned structural colours in visible and near-infrared regions by the industrially-scalable approach based on inherent resonant scattering inside the lattice. A facile-scalable method for the fabrication of elastomeric opal films with the abilities of light, temperature, and mechanical sensitivity that are potentially applied in rewritable optical data storage as well as used for promising tunable optoelectronic laser action materials has been realised by Gallei’s group [25]. In this work, we propose the use of a novel material for traffic warning displays in both sunny and rainy days. The previously published literatures have provided evidence that Poly(St-MMA-AA) copolymer photonic crystal arrays exhibited brilliant structural colours after a self-assembly process [18,26,27,28,29], which was generally synthesised by emulsion polymerisation that was based on the kinetic principles of free radical-initiated vinyl addition polymerisation [30]. There have been a few attempts at the study of achieving the stimuli-responsive behaviour of the photonic crystal films for amplifying their potential applications as sensing materials [31,32,33]. Manners and Ozin’s group [34] also developed an inverse polymer-gel opal with high electroactive property, and it could exhibit full colour at quiet low drive voltages. Carbon-based materials have promising applications in optoelectronic area, especially graphene-based materials [35,36,37]. Wang’s [38] and Zhang’s [39] group have successfully fabricated structurally coloured fibres via electrophoretic deposition on conductive carbon fibres by the magnetically induced self-assembly method. Carbon fabric is defined as a carbon-based textile material that consists of stacked crystalline graphitic layers. The crystalline structure provides the features of black colour appearance with improved mechanical properties, high thermal resistance, high electrical conductivity, and chemical stability [40]. The optical absorption of incoherent scattering of light by carbon fabrics covers the whole visible spectral region and further blocks the colour reflected by dye or pigment molecules. Meanwhile, the chemically inert surface of carbon fabric reveals poor affinity with dye in colouration process. Therefore, it is a great challenge to produce colour on carbon fabric through surface modification. Among current surface treatment approaches, plasma processing is regarded as an effective, economical, and time-saving surface modification method for altering the wettability of the materials [41]. It has been employed for surface modification of surface wettability to increase the affinity between the photonic crystal films and the carbon fabric substrates. However, there has been very limited attempts to adopt well-defined artificial photonic crystal patterning on carbon fabrics. Herein, we reported an available strategy for generating Poly(St-MMA-AA)/PCFs via the thermal assisted gravity sedimentation approach that can accelerate the accumulation of colloidal Poly(St-MMA-AA) nanoparticles in suspension through heating evaporation-induced assembly. Poly(St-MMA-AA) copolymer was synthesised and employed to establish photonic crystal films on carbon fabric substrates via the self-assembly approach. Tunable vivid structural colours were obtained by regulating the diameters of Poly(St-MMA-AA) nanoparticles during emulsion polymerisation. Colour appearance and resistivity discrepancy of Poly(St-MMA-AA)/PCFs was tested for applying in sunny- and rainy-day warning displays.

## 2. Materials and Methods

### 2.1. Materials and Chemicals

Plain-weave carbon fabrics (density 147 g/m^2^; weave count: warp 49 per inch; weft 47 per inch) that were made of 7.5 microns-diameter filament yarn were provided by Fuel Cell Store, College Station, TX, USA. Styrene (St), methyl methacrylate (MMA), acrylic acid (AA), sodium dodecyl sulfate (SDS), ammonium bicarbonate (≥99.0%, ReagentPlus^®^), and ammonium persulfate (APS, ≥98.0%, ACS reagent) were purchased from Tin Hang Technology Ltd., Hong Kong SAR, China; deionised water (H_2_O) was distilled by a Thermo Scientific GenPure Standard Water Purification System, Langenselbold, Hesse, Germany. All chemicals and materials in this work were used as received from their venders, without any further purification.

### 2.2. Preparation and Purification of Poly(St-MMA-AA) Nanoparticles

**Scheme 1:**
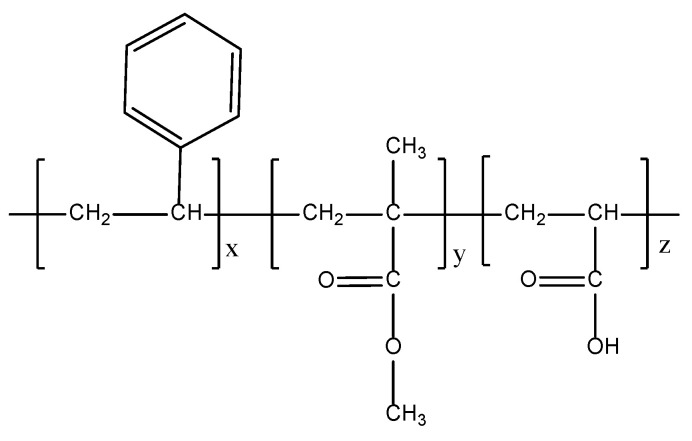
Chemical structure of Poly(St-MMA-AA) nanoparticle.

The experimental arrangement and method used for preparing Poly(St-MMA-AA) were similar to the previously reported emulsion polymerisation method [30]. Styrene (St) (20 g), methyl methacrylate (MMA) (1 g), acrylic acid (AA) (1 g), deionised water (100 g), sodium dodecyl sulfate (SDS) as an emulsifier (0.001–0.006 g), and then ammonium bicarbonate as a buffer (0.5 g) were sequentially added into a three-necked flask that was equipped with Nitrogen (N_2_) atmosphere, a reflux condenser, and a magnetic stirrer at a stirring speed of 500 rpm. The reaction mixture was kept at a constant temperature (70 °C) controlled by Lab Companion RW3-0525 Refrigerated & Heating Bath Circulator (Hong Kong Labware Co., Ltd., Hong Kong SAR, China) for 30 min, and then heated to 80 °C, followed by the addition of an aqueous APS solution prepared by dissolving 0.48 g APS in 15 g deionised water. Polymerisation was carried out for 10 h with continuous stirring. The resulting colloidal P(St-MMA-AA) nanoparticles were purified several times by centrifugation at 14,000 rpm for 75 min at 22 °C and then re-dispersed in deionised water by ultrasonication. The schematic chemical structure of as-synthesised Poly(St-MMA-AA) is depicted as Scheme 1.

### 2.3. Generation of Poly(St-MMA-AA) Photonic Crystal Films on Carbon Fabrics

**Scheme 2:**
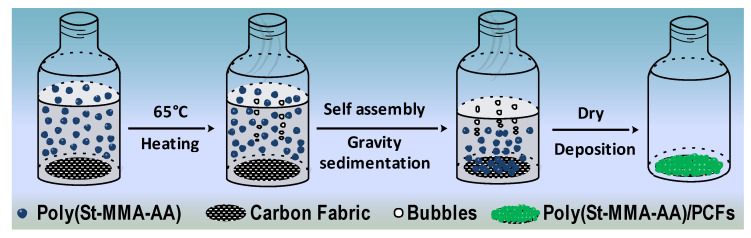
Schematic diagram of Poly(St-MMA-AA)/PCFs preparation process by thermal assisted gravity sedimentation method.

The stock solution of 8 wt.% Poly(St-MMA-AA) nanoparticles in suspension was used for preparing Poly(St-MMA-AA) photonic crystal films on carbon fabrics via the thermal assisted gravity sedimentation method, as shown in Scheme 2. A circular piece of fabric with a diameter of 2 cm was pre-added into a 20 mL glass bottle, followed by the addition of 2 mL nanoparticle suspension solution. Subsequently, the bottle was placed in a constant hot environment at 65 °C in a dry oven (Esco Isotherm^®^ Forced Convection Laboratory oven that was purchased from Hong Kong Labware Co., Ltd., Hong Kong SAR, China) for heating treatment. After 12 h of gravity sedimentation and self-assembly, the solvents were evaporated, the particles got assembled in a closer arrangement, and the Poly(St-MMA-AA) photonic crystal films were finally formed on carbon fabrics, hereafter referred to as Poly(St-MMA-AA)/PCFs. Wet Poly(St-MMA-AA)/PCFs was acquired by dipping treatment into a beaker with 500 mL H_2_O for two seconds. Besides, carbon fabrics were pre-treated by a plasma system (Surfx technologies, Redondo Beach, CA, USA) before thermal-assisted gravity sedimentation. The samples were laid on a flat plate and the operating temperature in the plasma reactor was 60 °C with the treatment condition of 0.4 mm/s operating speed, 290 W power, and input oxygen and helium flow rate were 0.4 L/min and 20 L/min, respectively.

### 2.4. Characterisation Methods

#### 2.4.1. Water-in-Air Contact Angle (WCA) Measurement 

Surface wettability properties of pristine and plasma treated carbon fabric were measured by SDS-350 Optical contact angle measuring instrument manufactured by Guangdong Dint Intelligent Technology Co., Ltd., Guangdong, China. The WCA measurement was performed by the method of applying a sessile of water that was placed on the surface of the specimens at room temperature. The WCA values were tested immediately after each droplet of 2 µL water touched the surface of the target samples. The WCA values of the plasma treated carbon fabrics were recorded by time (0, 5, 10, 15, 20, 22, 22, 24, and 26 min). 

#### 2.4.2. Microscopic Observation

Transmission electron microscopy (TEM) morphologies of Poly(St-MMA-AA) nanoparticles were detected by JEOL JEM-2011 Transmission Electron Microscope. Prior to conducting TEM measurement, Poly(St-MMA-AA) nanoparticles were first pre-dispersed and diluted in aqueous solution to approximately 100 ppm, 10 μL nanoparticle solution were then deposited on 400-mesh carbon-coated copper (Cu) grids, and the sample was completely air-dried prior to TEM observation. 

#### 2.4.3. Particle Size and Polydispersity Indices (Ð) Measurement

The average colloidal Poly(St-MMA-AA) particle sizes and polydispersity indices of the as-synthesised silica nanoparticles in suspension were measured and calculated by a particle size analyser provided from Zeta Plus, Brookhaven Instruments Corp., Holtsville, NY, USA. Solid-state particle sizes of Poly(St-MMA-AA), Poly(MMA-AA) Shell, Polystyrene (PS) core were measured from TEM images while using Nano measurer software, version 1.2, Fudan University, Shanghai, China.

#### 2.4.4. Optical Image Acquisition

Optical microscopic images of Poly(St-MMA-AA)/PCFs were examined using Leica M165 FC optical microscopy (Leica Microsystems, Newcastle, UK) integrated with 16.5:1 zoom optics. The images were captured at room temperature. 

The photographs of Poly(St-MMA-AA)/PCFs were taken with a 12-megapixel rear camera with a 28 mm wide-angle lens and f/1.8 aperture. The images were acquired in a light chamber under D65 illumination source. No adjustment of colour, pixels, and contrast was applied to these pictures. 

#### 2.4.5. Fourier Transform Infrared (FT-IR) Spectroscopy 

FT-IR spectrum with wave numbers from 400 cm^−1^ to 4000 cm^−1^ was obtained by subtracting the substrate effect using Thermo Scientific NicoletTM 380 FT-IR Spectrometer (Thermo Fisher Scientific, Waltham, MA, USA) to analyse the inherent chemical information of Poly(St-MMA-AA) nanoparticles. The setting number of scans was 64 and the resolution was 4 with 1.929 cm^−1^ data spacing.

#### 2.4.6. Raman Spectroscopy 

Micro-Raman spectrum ranged from 3200 to 300 cm^−1^ and it was measured by the Renishaw InVia Micro-Raman Spectroscopy system (Renishaw plc, Gloucestershire, UK) equipped with a confocal optical microscope for fast mapping. The Raman wavelength range was between 200 and 2200 nm, the spectral data was collected with resolution of 0.3 cm^−1^, and the stability was <±0.001 cm^−1^. The tested samples were irradiated with diode laser with excitation wavelength of 532 nm. The output power of laser was 5 mW, The Raman scatterings from the samples were collected by an objective lens, filtered into the microscope frame, and directed to a 50× eyepiece for visual observations. A multichannel charge-coupled device (CCD) detector was used for spectral analyses, and a digital camera with high sensitivity mode was applied for imaging. 

#### 2.4.7. Colour Property Measurement

Colour reflectance of Poly(St-MMA-AA)/PCFs was performed by an SF650 DataColour spectrophotometer from DataColor International (Lawrenceville, NJ, USA) with the D65 illumination source, and the aperture size was 9 mm. Reflectance in the wavelength in the range from 400 to 700 nm were obtained. Each datum was acquired by calculating the average value of four repeated measurements. The CIE L*a*b* values of the Poly(St-MMA-AA)/PCFs samples before and after dipping were tested by Datacolour ColourReader Pro portable spectrophotometer with 8 high CRI, white LED’s light source, and OLED (Organic Light Emitting Diodes) display.

#### 2.4.8. Electrical Resistivity Measurement

The surface resistivity of the specimens (carbon fabrics and wet Poly(St-MMA-AA)/PCFs) with smaller resistivity was measured by a four-probe method at room temperature with ST-2258A multifunction digital four-probe tester that was equipped with ST2558B-F01 Linear square resistance four probe (Suzhou Jingge Electronic Co., Ltd., Jiangsu, China), whereas the resistance of Poly(St-MMA-AA)/PCFs samples with higher resistivity was tested by FLUKE 15b+ digital multimetre (Fluke Corp., Washington, DC, USA) with the assistance of two copper electrodes at both ends of the tested sample. The final surface resistivity or resistance was obtained by performing five replications at different locations on the sample and the average value was calculated for analysis. The thickness of the samples was tested by SanLiang digital indicators, Sanliang Measuring Tool Co., Ltd., Dongguan, China.

## 3. Results and Discussion

### 3.1. WCA Analysis of Carbon Fabrics

**Figure 1:**
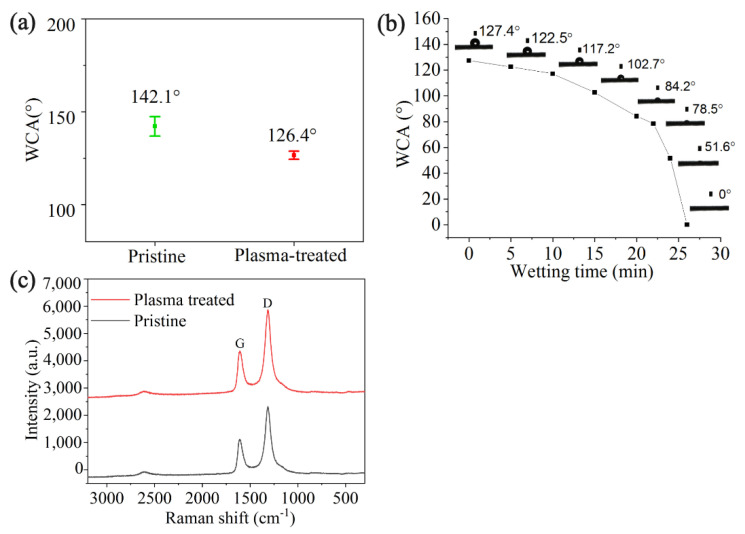
(**a**) The mean contact angle of carbon fabric before (in green) and after plasma treatment (in red); (**b**) A set of water contact angles (WCA) changes on plasma-treated carbon fabrics as a function of wetting time: 0, 5, 10, 15, 20, 22, 24, and 26 min; and, (**c**) Raman spectra of pristine carbon fabric and plasma treated carbon fabric.

**Figure 2:**
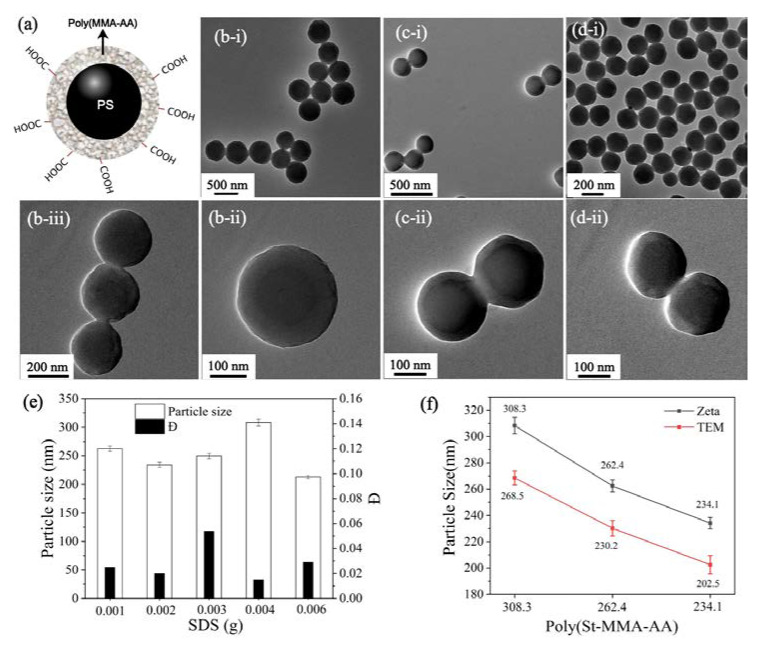
(**a**) Schematic representation of the structure of Poly(St-MMA-AA) nanoparticle with PS core and Poly(MMA-AA) shell structure; (**b**–**d**) transmission electron microscopy (TEM) images of Poly(St-MMA-AA) with various hydrodynamic diameters of (**b**) 308.3 nm, (**c**) 262.4 nm and (**d**) 234.1 nm with different magnification of (**b**-**i**) 5000×, (**b**-**ii**) 40,000×, (**b**-**iii**) 20,000×, (**c**-**i**) 8000×, (**c**-**ii**) 30,000×, (**d**-**i**) 10,000×, (**d**-**ii**) 30,000×; (**e**) different Poly(St-MMA-AA) particle sizes of 262.4, 234.1, 249.7, 308.3, and 213.1 nm successively with different Ð values of 0.025, 0.020, 0.054, 0.015, 0.029, as a function of sodium dodecyl sulfate (SDS) amount of 0.001 g, 0.002 g, 0.003 g, 0.004 g, and 0.006 g, respectively (from Zeta particle sizer); (**f**) particle size contrast of Poly(St-MMA-AA) nanoparticles measured by Zeta analyser and by Nano measurer 1.2 software from TEM micrographs.

Water contact angles (WCA) of both pristine and plasma-treated carbon fabrics were collected, as depicted in Figure 1a, and the result shows that the plasma-treated carbon fabrics with a mean contact angle of 126.4° had improved surface wettability, approximately 16° of angle lower than the pristine carbon fabric with contact angle reaching 142.1° on average, which demonstrates that the initial infiltration of plasma-treated carbon fabrics into colloidal suspension would be faster than pristine carbon fabrics, and plasma treatment with the function of increasing wettability contributes to making carbon fabrics become more promising candidates as the substrates for the formation of photonic crystals on them through thermal assisted gravity sedimentation. From Figure 1b, a set of contact angles of the plasma treated carbon fabric decreasing by time has been recorded, which shows that during the first 15 min, contact angles of carbon fabric had no obvious changes. As more time lapsed, the contact angle reached 0° after 26 min of absorption, which indicated that the carbon fabric was totally infiltrated. The complete infiltration time (26 min) of plasma-treated carbon fabrics is extremely shorter than the time of sedimentation process (12 h), which might cause a slight effect on the interactions between colloidal nanoparticles in suspension and the plasma-treated substrates. It has been proved from the result of contact angle test that the carbon fabrics would become more hydrophilic than the untreated one, and plasma treatment tends to be favourable for the formation of stronger binding force between the plasma-treated carbon fabrics and the colloidal nanoparticles with surrounding hydrophilic group (-COOH) on the surface of shell part, as presented in Figure 2b.

Raman spectroscopy was utilised in order to elucidate the nanostructures of pristine and plasma-treated carbon fabrics (Figure 2c) it can be found that there is no obvious change in the surface structure of carbon fabric before and after plasma treatment; however, the spectrum shows two major changes in the peak shifts of 1604 cm^−1^ (G peak) and 1314 cm^−1^ (D peak). The peak located at 1604 cm^−1^ represents the typical graphite crystallite structure, which belongs to the in-plane bond stretching of carbon atom, while the peak with higher intensity at 1314 cm^−1^ refers to the disordered carbon structure, corresponding to the vibration of crystallite component.

### 3.2. TEM Observation and Particle Size Analysis

The schematic structure of Poly(St-MMA-AA) nanoparticle is simulated, as depicted in Figure 2a, and it has been proved by TEM observations (Figure 2b-i,c-i,d-i) that the structure of Poly(St-MMA-AA) is in spherical/concentric circles shape with rigid polystyrene (PS) in the core and relatively soft Poly(MMA-AA) in the shell with the enfolding of carboxyl (-COOH) group generated from the acrylic acid component. The overview TEM images of Poly(St-MMA-AA) with different diameters of 308.3 nm, 262.4 nm, and 234.1 nm in Figure 2b–d suggest the existence of adhesive interaction at the contact points between adjacent nanoparticles and the introduction of Poly(MMA-AA) shell leads to better spontaneous arrangement of nanoparticles due to the adhesiveness and inter-particle interaction of Poly(MMA-AA) of nanoparticles. The shapes of Poly(St-MMA-AA) become less spherical as the peripheries are conjoined with deformation under inter-particle force between the neighbouring particles. The photonic bandgap showing brilliant structural colours is attributed to the high refractive index. The effective refractive index contrast of the Poly(MMA-AA) nanoparticles as building blocks and the media (air) in the interspace of the neighbouring nanoparticles, as well as the particle size (diameter) of nanoparticles, are the significant determinants of display of structural colours on Poly(St-MMA-AA)/PCFs. The presence of emulsifier sodium dodecyl sulfate (SDS) in the polymerisation process contributes to the formation and increasing proportion of photonic crystal array [42]. In this work, SDS as the variate with weight ratios of 0.001 g to 0.006 g has been added to generate diverse particle sizes. In the present investigation, variations of particle size by the change of SDS amount were analysed in Figure 2e. The polymerisation does not start in the emulsifier (SDS) micelles, but in the aqueous phase after initiating of the initiator (APS), followed by the formation of radicals, according to the micellar mechanism of the particle formation by emulsion polymerisation as elucidated before (comprise particle nucleation and growth stage). Primary charged polymer particles are generated by precipitation of oligomer radicals, while they are saturated after reaching the critical micelle concentration (CMC) according to Fitch and Tsai’s findings [43,44], which combines with other soluble radicals again to form the neutral surface active substances, and finally flocculating appears as the reaction of the other radicals with the solubility is terminated [45]. SDS emulsifier contributes to dispersing and stabilising of the particles and it also leads to an increase in the solubility of the radicals during the above formation [46]. There was a hydrogen bond that formed between SO_4_^−^Na^+^ of SDS and –COOH group (see Figure 2b) surrounded on the particle peripheries, which is conducive to encapsulating around the particle in order to provide a stable emulsion environment. Hansen et al. [47] showed the decreasing number of new particles was due to the decrease of free emulsifier (SDS) concentration beneath CMC, which implies that the increasing concentration of SDS would increase the new particle number contributing to form larger particle aggregates. Accordingly, the growth trend of Poly(St-MMA-AA) nanoparticle size was induced on the whole with the increasing weight ratios of SDS from 0.001 g to 0.006 g that caused a highly number of primary particles and consequently resulted in smaller particle size, along with the Poly(St-MMA-AA) hydrodynamic particle size decreasing from 308.3 nm to 213.1 nm. Figure 2f allows for a comparison of particle sizes (diameter) of Poly(St-MMA-AA) by both the particle size analyser and TEM micro-images. It is notable that the average particle sizes of three-size nanoparticles that were measured from TEM micro-images were approximately 30 nm smaller than that obtained using Zeta analyser. The different results of particle sizes are attributable to the discrepancy of the nanoparticle measuring environment; in other words, Brownian dynamics of Poly(St-MMA-AA) colloidal nanoparticles with hydrodynamic interactions in the aqueous dispersion and they impeded the accurate measurement of particle size when tested using Zeta particle size analyser, and the particles were in swollen form in aqueous medium, which resulted in the larger particle size result. The particle samples were pre-treated by drying in order to achieve the removal of media, which results in the shrinkage of nanoparticles as compared to that from Zeta analyser and it is proposed to obtain the decrease of particle size after water evaporation.

### 3.3. Chemical Characteristics by FT-IR and Raman Spectroscopy

**Figure 3:**
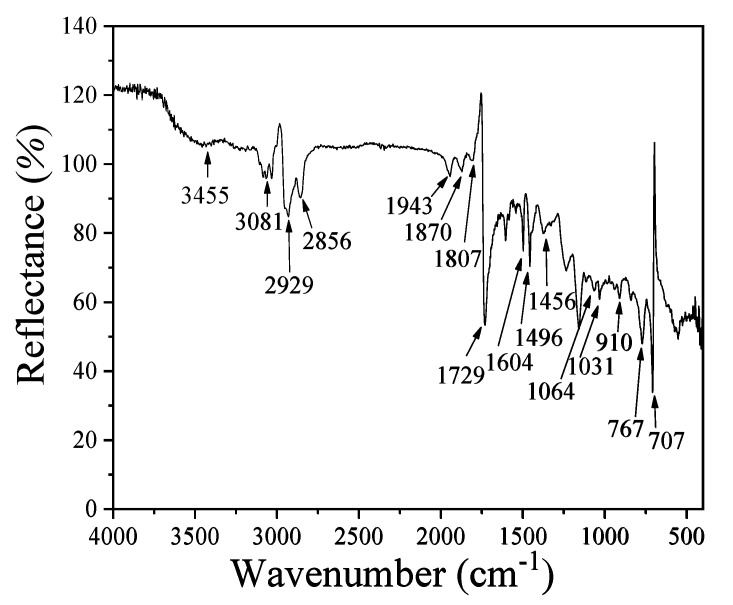
Fourier Transform Infrared (FT-IR) spectrum of the as-synthesised core/shell Poly(St-MMA-AA) nanoparticles (308.3 nm).

Figure 3 illustrates the FT-IR spectrum of 308.3 nm-diameter Poly(St-MMA-AA) nanoparticles. The stretching vibration of the hydroxyl (O–H) which comes from –COOH group causes the reflectance peak at 3455 cm^−1^. The positions at 3081 cm^−1^ represent the vibrations of the methyl carbon (–CH_3_) group. The presence of a sharp prominent band at 1729 cm^−1^ suggests a good possibility of the existence of carbonyl (C=O) stretching from –COOH functional group in the shell part of the copolymer [48,49]. The characteristic peaks that are observed in the fingerprint region at 1031 cm^−1^ and 910 cm^−1^ wavenumbers are attributed to C–O–C symmetric stretching vibration, which is associated with the C-COOCH_3_ group of MMA located at the shell region of nanoparticles [50], the intensities of these peaks are weak because of the small amount of MMA monomer involved in the synthetic process, while the peak at 1604 cm^−1^ can be associated with –C=C stretching vibrations of aromatic double bond in styrene molecule. The several peaks at 1943 cm^−1^, 1870 cm^−1^, and 1807 cm^−1^ in the region of 1800–2000 cm^−1^ are expected for weak overtone and combination bands of mono-substituted aromatic rings [51,52,53]. The signal corresponding to the deformation of C–H bonds in the methyl group is confirmed at 1456 cm^−1^. These vibrational peaks indicate the existence of benzene rings in the core segment of as-synthesised nanoparticles. The pronounced peaks at 2929 cm^−1^ and 2856 cm^−1^ are obtained due to the asymmetric and symmetric stretching vibration of aliphatic C-H bond of CH_2_, and flexion of C–H in the plane contribute to the presence of 1064 cm^−1^ wavenumber [54]. The peaks generating at 767 cm^−1^ and 707 cm^−1^ are significantly associated with the flexural vibrations of C–H group. The results of FT-IR analysis demonstrate the existence of each chemical functional group of copolymer Poly(St-MMA-AA), and the spectra provides confirmatory evidence that monomer styrene (St), methyl methacrylate (MMA), and acrylic acid (AA) have incorporated with each other to produce Poly(St-MMA-AA) through emulsion polymerisation reaction, which is also consistent with experimental observation from Lai’s group reported in the literature [55]. 

**Figure 4:**
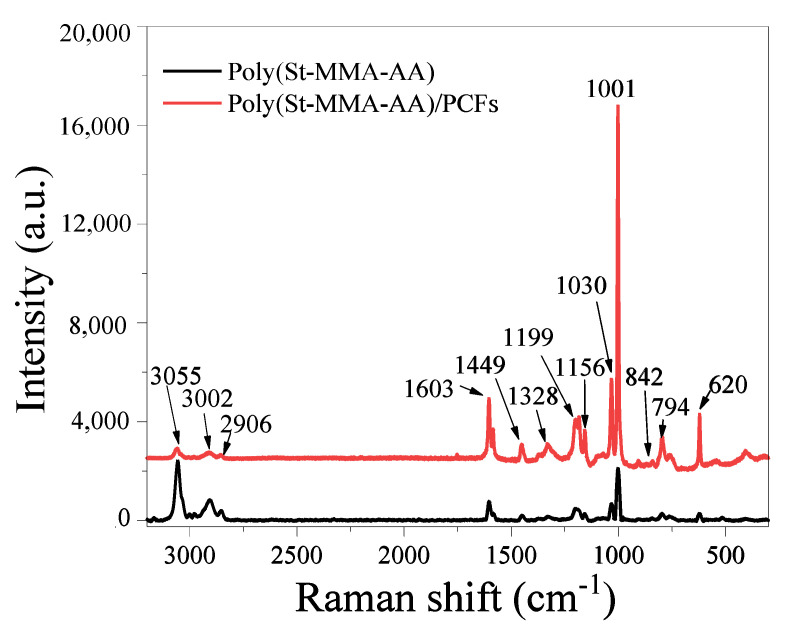
Raman spectra of 308.3 nm-diameter Poly(St-MMA-AA) nanoparticles prepared on aluminium substrate adhering to the surface of a coverslip and Poly(St-MMA-AA)/PCFs made of 308.3 nm-diameter Poly(St-MMA-AA) nanoparticles. The laser focus is centred within the nanoparticle agglomerates.

The surface structure change of 308.3 nm-diameter Poly(St-MMA-AA) copolymer with various polymeric components and Poly(St-MMA-AA)/PCFs were further analysed by Raman spectroscopy and the spectrum was recorded, as in Figure 4. Based on the assignment of Raman active lines, the characteristic peak that was detected at 3055 cm^−1^ was due to aromatic C–H stretching vibration referring to the core information of the copolymer [56]. The peak at 2906 cm^−1^ indicating the stretching of –CH_2_ or C–H group can be found. The peaks at 842 and 1199 cm^−1^ represent C–COOH stretching and C–O stretching coupled with in-plane bending related to shell composition, respectively [57]. Raman scattering from the aromatic ring skeletal stretch and ring deformation mode about the core part of the copolymer Poly(St-MMA-AA) is also detectable at 1603 and 620 cm^−1^ [58]. Poly(St-MMA-AA)/PCFs shows a stronger peak at 1603 cm^−1^ when compared with Poly(St-MMA-AA) nanoparticles, mainly due to the effect of crystallite structure of carbon fabric substrate. It is notable that the typical one at 1001 cm^−1^ showing a strong marker is the most prominent, which is due to the breathing mode of aromatic ring that had an intense aromatic carbon-carbon bond stretch [58,59]. It is related to the polystyrene core of the Poly(St-MMA-AA) nanoparticle. The characteristic band at 794 cm^−1^ is the vibrational mode of phenyl rings group that are present in the core segment of the copolymer [59]. 1030 cm^−1^ corresponds to C–H in-plane deformation, 1156 cm^−1^ is C–C stretch, and the peak appearing at 1449 cm^−1^ indicates CH_2_ scissoring of core (PS) composition [58,60]. The Raman spectra also show the characteristic band at 3002 cm^−1^ and this band indicates the vibration of C–H of O–CH_3_, while the peak at 1328 cm^−1^ confirms the vibration of C–COO group, which indicates the presence of Poly(MMA-AA) shell composition [61]. The presence of the intrinsic chemical information of Poly(St-MMA-AA) copolymer could be ascertained with the observation from the combination of Raman and FT-IR analysis.

### 3.4. Colour Measurement

**Figure 5:**
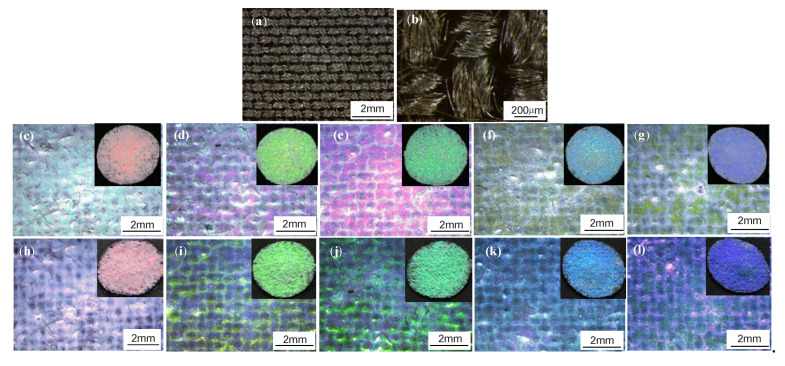
Optical microscope images of plain-weave carbon fabrics with magnification of (**a**) 160× and (**b**) 1000×. (**c**–**g**) Optical micrographs of Poly(St-MMA-AA)/PCFs before dipping treatment. (Particle diameters from (**c**) to (**g**): 308.3, 262.4, 249.7, 234.1, and 213.1 nm). (Magnification: 160×). (**h**–**l**) The corresponding optical micrographs of poly(St-MMA-AA)/PCFs after dipping treatment. (Particle diameters from (h) to (l): 308.3, 262.4, 249.7, 234.1, and 213.1 nm). Magnification for (**c**) to (l): 160×). The insert showed corresponding optical images of carbon fabrics deposited with poly(St-MMA-AA) particle with diameters of 308.3, 262.4, 249.7, 234.1, and 213.1 nm.

**Figure 6:**
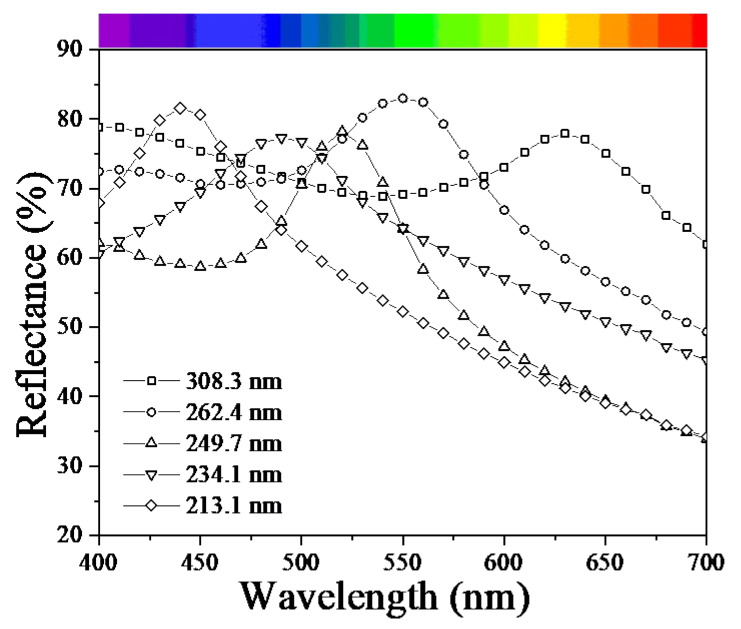
Reflectance spectra of Poly(St-MMA-AA)/PCFs showing different colours by the changes of Poly(St-MMA-AA) particle sizes (308.3, 262.4, 249.7, 234.1, and 213.1 nm).

Figure 5a,b exhibit the microstructure of the plasma treated plain carbon fabrics with magnified images. From the optical micrographs from Figure 5c–g, it is clear that the voids between carbon fibres generated from the texture of fabric and the arrangement of filaments were filled with self-assembled Poly(St-MMA-AA) nanoparticles. The intermolecular van der Waals force regarded as dominant interaction energy among the adjacent Poly(St-MMA-AA) nanoparticles in the coverage area contributes to the formation of self-assembled photonic crystal films [62,63] and, thereby, Poly(St-MMA-AA)/PCFs with vivid structural colours were constructed. The variations of colours were primarily determined by the Poly(St-MMA-AA) nanoparticle sizes. In optical micrographs, the colours had a red hue when the particle size decreased to 249.7 nm and then turned to a green hue with a further decrease of particle diameter. The inserts in Figure 5c–g shows the photograph of Poly(St-MMA-AA)/PCFs with the structural colours (salmon, chartreuse, springgreen, skyblue, mediumpurple) (left to right) as the Poly(St-MMA-AA) particle diameters changed from 308.3 nm to 213.1 nm. This is in good agreement with experimental results of reflectance, as displayed in Figure 6. The resulting reflected wavelength with the maximum amplitude of specific wavelength (λmax) at 650, 570, 520, 490, and 440 nm can be predicted by the Bragg’s and Snell’s laws [64,65], commensurate to different Poly(St-MMA-AA)/PCFs differing from particle size of 308.3, 262.4, 249.7, 234.1, and 213.1 nm, which produce brilliant colours from salmon, chartreuse, spring green, skyblue to medium purple, a blue-hue occurs in the spectra would occur. The theoretical diffraction peak position of the spectra (λi) could be empirically calculated as Equation (1) [55,66], as the lattice was in close-packed structure (face-centred cubic, fcc),

(1) $$\lambda i={\left(\frac{\pi }{3\sqrt{2}f_{P}}\right)}^{1/3}{\left(\frac{8}{3}\right)}^{1/2}D_{Pi}\;{\left(n_{eff}{}^{2}-sin^{2}\theta \right)}^{1/2}$$

where, $D_{Pi}$ is the diameter of Poly(St-MMA-AA) nanoparticle, $f_{P}$ is the filling ratio of the nanoparticles (the value is 0.74 in fcc [26]), θ means the normal incident angle (θ = 0°, the direction of incident light was perpendicular to the ordered photonic crystal film), and $n_{eff}$ is the effective refractive index of the photonic crystal film structure that could be obtained from Equation (2) [65],

(2) $$n_{eff}={\left[n_{P}{}^{2}f_{P}+n_{m}{}^{2}\left(1-f_{P}\right)\right]}^{1/2}$$

where, $n_{P}$ is the refractive index of Poly(St-MMA-AA) nanoparticle ($n_{P}=1.5916$) and $n_{m}$ is the refractive index of air ($n_{air}=1$).

For instance, particle size of Poly(St-MMA-AA)/PCF with salmon colour, the particle size measured in suspension (by Zeta) was 308.3 nm, while the particle size in dry state for TEM observation was 268.5 nm (as shown in Figure 2d), which is taken for Bragg calculation according to the formula of Equation (1) and Equation (2); hence, λi can be obtained with the value of 640.6 nm. The experimental data with photonic bandgap position (λmax = 650 nm), as mentioned above, do not deviate appreciably from this theoretical result of λi. The ultimate surface cracks, defects, and loose-packed structures of the Poly(St-MMA-AA) photonic crystal film on carbon fabrics, as presented in Figure 5c–g, should be responsible for the subtle difference (less than 10 nm) between the experimental and theoretical results. The enlarged lattice interlayer spacing of the crystalline planes would cause the increase of diffraction peak wavelength if it was not densely-packed structure, resulting in a numeric value λmax of the experimental value greater than the theoretical one λi. Therefore, a red shift from the theoretical value to experimental value could appear, which matches the findings reported in extant literature [55,65]. As the peak wavelengths refer to the colours of Poly(St-MMA-AA)/PCFs in visible spectrum at the top of reflectance curve profile in Figure 6, the simulative colours could be estimated by assessing from the diameters of as-synthesised Poly(St-MMA-AA) nanoparticles, since the colour change by calculation is in accordance with the perceptual colour difference that is shown in the insert of Figure 5c–g. Figure 5h–l show photos of the corresponding samples after dipping treatment. The colours of the insert images were enhanced. Since water medium was filled in the lattice spacing, $n_{m}\;$ in this situation should be the refractive index of water larger than that of air, which swells the periodic interplanar spacing, and the bandgap position is increased. Consequently, a red shift of colours is caused by dipping treatment and the colour difference that existed between Poly(St-MMA-AA)/PCFs in the dry state and wet state.

The colour difference between Poly(St-MMA-AA)/PCFs with the adjacent nanoparticle sizes can be analysed by Commission Internationale de l’Eclairage (*CIE*) L*a*b* coordinates (Table 1), where L*, a*, b* correspond to the degree of lightness, red/green coordinates, yellow/blue coordinates to investigate the effect of variations of Poly(St-MMA-AA) diameters on the ultimate structural colour. *CIE* colourimetry could intuitively reflect the colour variation and decode the colours with quiet precise data. Parameter Δa* represents the discrepancy in red and green, from 308.3 nm to 249.7 nm, the value of Δa* is negative (−12.73, −10.78), which indicates that the structural colour became greener along with the decrease of nanoparticle sizes. Conversely, the colour turned to be redder (positive values of Δa*: 9.86, 7.49) with the further decrease of particle diameters. Similarly, for Δb*, the positive and negative values refer to yellower and bluer change in structural colour, respectively. Consequently, as the particle sizes decreased from 308.3 nm to 213.1 nm, the colour first turned from yellowish to blueish and eventually became yellow shift. The total colour difference ΔE_ab_^*^ between every two adjacent colours has been given as Equation (3) [67], in which ΔE_ab_^*^ derived from the difference of lightness ΔL* and the values of Δa*, Δb*. The calculated data have numerically proved the colour difference that exists in adjacent sized Poly(St-MMA-AA)/PCFs.

(3) $$\Delta E_{ab}{}^{*}=\sqrt{{\left(\Delta L^{*}\right)}^{2}+{\left(\Delta a^{*}\right)}^{2}+{\left(\Delta b^{*}\right)}^{2}}$$

### 3.5. Electrical Resistivity of Poly(St-MMA-AA)/PCFs

**Figure 7:**
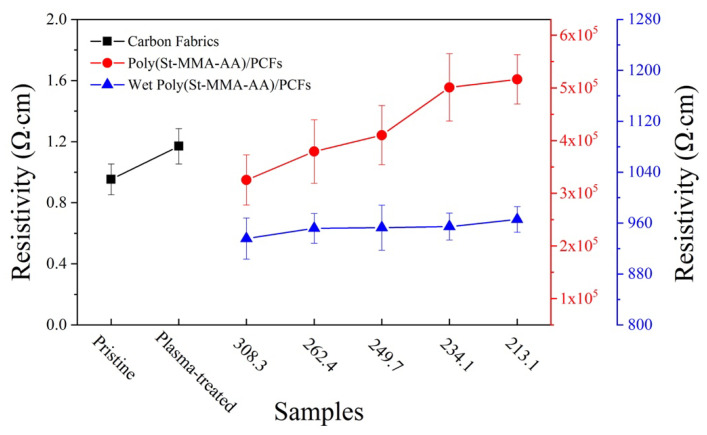
Electrical resistivity of pristine, plasma-treated carbon fabrics, Poly(St-MMA-AA)/PCFs and wet Poly(St-MMA-AA)/PCFs with various diameters of 308.3, 262.4, 249.7, 234.1 and 213.1 nm.

The electrical resistivity of pristine, plasma-treated carbon fabric, Poly(St-MMA-AA)/PCFs, and wet Poly(St-MMA-AA)/PCFs obtained by dipping treatment with different particle sizes has been measured, as shown in Figure 7. After plasma treatment, surface wettability of carbon fabrics is significantly improved, but the coarse surface that is induced by plasma slightly reduces the conductivity of carbon fabrics and simultaneously increase the resistivity from 0.953 to 1.170 Ω·cm. The experimental results also showed that the resistivity was obviously increased after deposition of Poly(St-MMA-AA) photonic crystal film on the carbon fabrics as compared to pure carbon fabrics, because the deposition of Poly(St-MMA-AA) nanoparticles on the surface of carbon fabrics and the regions inside the cracks, defects, and spacing among the carbon fibre filaments impeded the conductive path, which resulted in the increase of resistivity by five order of magnitudes. As the size of Poly(St-MMA-AA) nanoparticle decreases from 308.3 nm to 213.1 nm, resistivities of Poly(St-MMA-AA)/PCFs before dipping treatment ranged from 3.25 × 10^5^ to 5.16 × 10^5^ Ω·cm. However, the corresponding resistivities after dipping treatment ranged from 9.36 × 10^2^ to 9.66 × 10^2^ Ω·cm. When considering the dipping treatment that is regarded as rainy days in our real life, the intrinsic decrease of resistivity on the wetting surface is attributed to water trapped at the free space between nanoparticles. The water indeed contributes to propagation of conductive path along the particle film. As particle diameter becomes smaller, the packing density becomes lower and vice versa, as illustrated from the graphical plots of resistivity vs. particle diameter in Figure 7. The resistivity difference for larger particle diameter such as 308.3 nm is smaller when compared to that with smaller particle diameter. In other words, smaller sized particles with lower particle packing density facilitate more free space or gap filled with water between adjacent particles, resulting in a large variation in resistivity difference between the dry and wet state. 

## 4. Conclusions

In conclusion, this study has successfully shown that preparation of Poly(St-MMA-AA)/PCFs with desirable vivid structural colours transforming remarkably from salmon to medium purple for the control of the Poly(St-MMA-AA) sizes by adjusting the quantity of the emulsifier is feasible. The concentric shape of Poly(St-MMA-AA) with Poly(MMA-AA) shell and PS core, as well as the chemical composition of the Poly(St-MMA-AA) copolymer, were attested. Explicitly speaking, the structural colours in the visible spectrum could be predicted and verified by the calculation from the sizes of as-synthesised Poly(St-MMA-AA) nanoparticles according to the Bragg’s and Snell’s laws. The colour difference of Poly(St-MMA-AA)/PCFs with adjacent sized Poly(St-MMA-AA) nanoparticles was measured by theoretical arithmetic with *CIE*) L*a*b* values. The obvious changes of surface resistivity among carbon fabrics, Poly(St-MMA-AA)/PCFs under dry and wet conditions have been investigated. The deposition of Poly(St-MMA-AA) photonic crystal films increased the resistivity of the carbon fabrics, and then decreased after dipping treatment. The proposed method provides Poly(St-MMA-AA)/PCFs the potential applications in smart displays for traffic warning signals, in spite of the large-scale fabrication of Poly(St-MMA-AA)/PCFs still remaining a challenge.

## Figures and Tables

**Table 1:** Colour coordinates (CIE L*, a*, b*) for Poly(St-MMA-AA)/PCFs as a function of Poly(St-MMA-AA) particle diameters.

Particle size (nm)	L* ^1^	a* ^2^	Δa*	Colour shift related to a*	b* ^3^	Δb*	Colour shift related to b*	ΔE_ab_*	
308.3	58.11	3.47	−12.73	Green shift	−4.07	6.44	Yellow shift	14.39
262.4	59.96	−9.26			2.32			
			−10.78	Green shift		−2.36	Blue shift	13.09
249.7	52.92	−20.04			−0.04			
			9.86	Red shift		−5.34	Blue shift	11.37
234.1	54.9	−10.18			−5.38			
			7.49	Red shift		26.50	Yellow shift	28.27
213.1	48.53	−2.69			−21.12			

^1^L*: Lightness (L*= 100 indicates white, L*= 0 yields black); ^2^a*: the red(+)/green(−) coordinate; ^3^b*: the yellow(+)/blue(−) coordinate.

## References

[1] Zhou L., Li Y., Liu G., Fan Q., Shao J. (2016). Study on the correlations between the structural colors of photonic crystals and the base colors of textile fabric substrates. Dyes Pigm..

[2] Liu G., Zhou L., Zhang G., Li Y., Chai L., Fan Q., Shao J. (2017). Fabrication of patterned photonic crystals with brilliant structural colors on fabric substrates using ink-jet printing technology. Mater. Des..

[3] Ge D., Lee E., Yang L., Cho Y., Li M., Gianola D.S., Yang S. (2015). A robust smart window: reversibly switching from high transparency to angle-independent structural color display. Adv. Mater..

[4] Lee H.S., Shim T.S., Hwang H., Yang S.M., Kim S.H. (2013). Colloidal Photonic Crystals toward Structural Color Palettes for Security Materials. Chem. Mater..

[5] Zhou C., Qi Y., Zhang S., Niu W., Ma W., Wu S., Tang B. (2020). Rapid fabrication of vivid noniridescent structural colors on fabrics with robust structural stability by screen printing. Dyes Pigm..

[6] Li Q., Zhang Y., Shi L., Qiu H., Zhang S., Qi N., Hu J., Yuan W., Zhang X., Zhang K.Q. (2018). Additive Mixing and Conformal Coating of Noniridescent Structural Colors with Robust Mechanical Properties Fabricated by Atomization Deposition. ACS Nano.

[7] Ding H., Zhu C., Tian L., Liu C., Fu G., Shang L., Gu Z. (2017). Structural Color Patterns by Electrohydrodynamic Jet Printed Photonic Crystals. ACS Appl. Mater. Interfaces.

[8] Chen S., Su M., Zhang C., Gao M., Bao B., Yang Q., Su B., Song Y. (2015). Fabrication of Nanoscale Circuits on Inkjet-Printing Patterned Substrates. Adv. Mater..

[9] Zeng Q., Ding C., Li Q., Yuan W., Peng Y., Hu J., Zhang K.Q. (2017). Rapid fabrication of robust, washable, self-healing superhydrophobic fabrics with non-iridescent structural color by facile spray coating. RSC Adv..

[10] Ishii M., Narita T., Hayata Y., Nishimura A., Tachi K. (2011). Simple and Rapid Fabrication of Large-Scale Polymer-Immobilized Colloidal Crystals by Spray Coating. Macromol. Mater. Eng..

[11] Gao W., Rigout M., Owens H. (2016). Self-assembly of silica colloidal crystal thin films with tuneable structural colours over a wide visible spectrum. Appl. Surf. Sci..

[12] Liu G., Zhou L., Wang C., Wu Y., Li Y., Fan Q., Shao J. (2015). Study on the high hydrophobicity and its possible mechanism of textile fabric with structural colors of three-dimensional poly(styrene-methacrylic acid) photonic crystals. RSC Adv..

[13] Liu Z., Zhang Q., Wang H., Li Y. (2013). Structurally colored carbon fibers with controlled optical properties prepared by a fast and continuous electrophoretic deposition method. Nanoscale.

[14] Katagiri K., Tanaka Y., Uemura K., Inumaru K., Seki T., Takeoka Y. (2017). Structural color coating films composed of an amorphous array of colloidal particles via electrophoretic deposition. NPG Asia Mater..

[15] Wu X., Hong R., Meng J., Cheng R., Zhu Z., Wu G., Li Q., Wang C.F., Chen S. (2019). Hydrophobic Poly(tert-butyl acrylate) Photonic Crystals towards Robust Energy-Saving Performance. Angew. Chem. Int. Ed. Engl..

[16] Fan W., Zeng J., Gan Q., Ji D., Song H., Liu W., Shi L., Wu L. (2019). Iridescence-controlled and flexibly tunable retroreflective structural color film for smart displays. Sci. Adv..

[17] Meng Y., Qiu J., Wu S., Ju B., Zhang S., Tang B. (2018). Biomimetic Structural Color Films with a Bilayer Inverse Heterostructure for Anticounterfeiting Applications. ACS Appl. Mater. Interfaces.

[18] Guo J., Li H., Ling L., Li G., Cheng R., Lu X., Xie A.Q., Li Q., Wang C.F., Chen S. (2020). Green Synthesis of Carbon Dots toward Anti-Counterfeiting. ACS Sustain. Chem. Eng..

[19] Kats M.A., Blanchard R., Genevet P., Capasso F. (2013). Nanometre optical coatings based on strong interference effects in highly absorbing media. Nat. Mater..

[20] Du X., Wang J., Cui H., Zhao Q., Chen H., He L., Wang Y. (2017). Breath-Taking Patterns: Discontinuous Hydrophilic Regions for Photonic Crystal Beads Assembly and Patterns Revisualization. ACS Appl. Mater. Interfaces.

[21] Gallei M. (2018). Functional Polymer Opals and Porous Materials by Shear-Induced Assembly of Tailor-Made Particles. Macromol. Rapid Commun..

[22] Finlayson C.E., Baumberg J.J. (2017). Generating bulk-scale ordered optical materials using shear-assembly in viscoelastic media. Materials.

[23] Zhao Q., Finlayson C.E., Snoswell D.R., Haines A., Schäfer C., Spahn P., Hellmann G.P., Petukhov A.V., Herrmann L., Burdet P. (2016). Large-scale ordering of nanoparticles using viscoelastic shear processing. Nat. Commun..

[24] Pursiainen O.L., Baumberg J.J., Winkler H., Viel B., Spahn P., Ruhl T. (2007). Nanoparticle-tuned structural color from polymer opals. Opt. Express.

[25] Schäfer C.G., Gallei M., Zahn J.T., Engelhardt J., Hellmann G.P., Rehahn M. (2013). Reversible light-, thermo-, and mechano-responsive elastomeric polymer opal films. Chem. Mater..

[26] Ding T., Smoukov S.K., Baumberg J.J. (2015). Stamping colloidal photonic crystals: a facile way towards complex pixel colour patterns for sensing and displays. Nanoscale.

[27] Li Q., Qi N., Peng Y., Zhang Y., Shi L., Zhang X., Lai Y., Wei K., Kim I.S., Zhang K.-Q. (2017). Sub-micron silk fibroin film with high humidity sensibility through color changing. RSC Adv..

[28] Yavuz G., Felgueiras H.P., Ribeiro A.I., Seventekin N., Zille A., Souto A.P. (2018). Dyed Poly(styrene-methyl Methacrylate-acrylic Acid) Photonic Nanocrystals for Enhanced Structural Color. ACS Appl. Mater. Interfaces.

[29] Zhu Z., Zhang J., Tong Y.-l., Peng G., Cui T., Wang C.-F., Chen S., Weitz D.A. (2018). Reduced Graphene Oxide Membrane Induced Robust Structural Colors toward Personal Thermal Management. ACS Photonics.

[30] Yuan W., Zhou N., Shi L., Zhang K.Q. (2015). Structural Coloration of Colloidal Fiber by Photonic Band Gap and Resonant Mie Scattering. ACS Appl. Mater. Interfaces.

[31] Schäfer C.G., Winter T., Heidt S., Dietz C., Ding T., Baumberg J.J., Gallei M. (2015). Smart polymer inverse-opal photonic crystal films by melt-shear organization for hybrid core–shell architectures. J. Mater. Chem. C.

[32] Arsenault A.C., Míguez H., Kitaev V., Ozin G.A., Manners I. (2003). A Polychromic, Fast Response Metallopolymer Gel Photonic Crystal with Solvent and Redox Tunability: A Step Towards Photonic Ink (P-Ink). Adv. Mater..

[33] Winter T., Su X., Hatton T.A., Gallei M. (2018). Ferrocene-Containing Inverse Opals by Melt-Shear Organization of Core/Shell Particles. Macromol. Rapid Commun..

[34] Puzzo D.P., Arsenault A.C., Manners I., Ozin G.A. (2009). Electroactive inverse opal: a single material for all colors. Angew. Chem. Int. Ed..

[35] Ahmadivand A., Gerislioglu B., Ramezani Z. (2019). Gated graphene island-enabled tunable charge transfer plasmon terahertz metamodulator. Nanoscale.

[36] Gerislioglu B., Ahmadivand A., Pala N. (2017). Hybridized plasmons in graphene nanorings for extreme nonlinear optics. Opt. Mater..

[37] Ahmadivand A., Gerislioglu B., Noe G.T., Mishra Y.K. (2019). Gated graphene enabled tunable charge–current configurations in hybrid plasmonic metamaterials. ACS Appl. Electron. Mater..

[38] Shang S., Liu Z., Zhang Q., Wang H., Li Y. (2015). Facile fabrication of a magnetically induced structurally colored fiber and its strain-responsive properties. J. Mater. Chem. A.

[39] Zhou N., Zhang A., Shi L., Zhang K.Q. (2013). Fabrication of Structurally-Colored Fibers with Axial Core–Shell Structure via Electrophoretic Deposition and Their Optical Properties. ACS Macro Lett..

[40] Frank E., Steudle L.M., Ingildeev D., Sporl J.M., Buchmeiser M.R. (2014). Carbon fibers: precursor systems, processing, structure, and properties. Angew. Chem. Int. Ed. Engl..

[41] Chu P.K., Chen J.Y., Wang L.P., Huang N. (2002). Plasma-surface modification of biomaterials. Mater. Sci. Eng. R Rep..

[42] Cong H., Cao W. (2003). Colloidal Crystallization Induced by Capillary Force. Langmuir.

[43] Fitch R.M., Prenosil M.B., Sprick K.J. (1969). The mechanism of particle formation in polymer hydrosols. I. Kinetics of Aqueous Polymerization of Methyl Methacrylate. J. Polym. Sci. Part. C Polym. Symp..

[44] Fitch R.M., Tsai C.H. (1971). Polymer Colloids.

[45] Aslamazova T.R. (1995). Emulsifier-free latexes and polymers on their base. Prog. Org. Coat..

[46] Litt M., Patsiga R., Stannett V. (1970). Emulsion polymerization of vinyl acetate. II. Polymer Chemistry. J. Polym. Sci. Part A -1.

[47] Hansen F.K., Ugelstad J. (1979). Particle nucleation in emulsion polymerization. III. Nucleation in systems with anionic emulsifier investigated by seeded and unseeded polymerization. J. Poly. Sci. Polym. Chem. Ed..

[48] Willis H.A., Zichy V.J.I., Hendra P.J. (1969). The laser-Raman and infra-red spectra of poly (methyl methacrylate). Polymer.

[49] Ifijen H.I., Ikhuoria E.U., Omorogbe S.O. (2019). Correlative studies on the fabrication of poly(styrene-methyl-methacrylate-acrylic acid) colloidal crystal films. J. Dispers. Sci. Technol..

[50] Thakur V.K., Vennerberg D., Madbouly S.A., Kessler M.R. (2014). Bio-inspired green surface functionalization of PMMA for multifunctional capacitors. RSC Adv..

[51] Olmos D., Martin E.V., Gonzalez-Benito J. (2014). New molecular-scale information on polystyrene dynamics in PS and PS-BaTiO3 composites from FTIR spectroscopy. PCCP.

[52] Qiu L., Chen W., Qu B. (2005). Structural characterisation and thermal properties of exfoliated polystyrene/ZnAl layered double hydroxide nanocomposites prepared via solution intercalation. Polym. Degrad. Stab..

[53] Gu R., Xu W.Z., Charpentier P.A. (2014). Synthesis of graphene-polystyrene nanocomposites via RAFT polymerization. Polymer.

[54] León-Bermúdez A.Y., Salazar R. (2008). Synthesis and characterization of the polystyrene-asphaltene graft copolymer by FT-IR spectroscopy. CT&F-Ciencia Tecnología y Futuro.

[55] Lai C.F., Li J.S. (2019). Self-assembly of colloidal Poly(St-MMA-AA) core/shell photonic crystals with tunable structural colors of the full visible spectrum. Opt. Mater..

[56] Watanabe K., Palonpon A.F., Smith N.I., Kasai A., Hashimoto H., Kawata S., Fujita K. (2015). Structured line illumination Raman microscopy. Nat. Commun..

[57] Dong J., Ozaki Y., Nakashima K. (1997). Infrared, Raman, and near-infrared spectroscopic evidence for the coexistence of various hydrogen-bond forms in poly (acrylic acid). Macromolecules.

[58] Bridges T.E., Houlne M.P., Harris J.M. (2004). Spatially resolved analysis of small particles by confocal Raman microscopy: Depth profiling and optical trapping. Anal. Chem..

[59] Fan Y., Cornelius C.J. (2013). Raman spectroscopic and gas transport study of a pentablock ionomer complexed with metal ions and its relationship to physical properties. J. Mater. Sci..

[60] Mazilu M., De Luca A.C., Riches A., Herrington C.S., Dholakia K. (2010). Optimal algorithm for fluorescence suppression of modulated Raman spectroscopy. Opt. Express.

[61] Xu X., Ming H., Zhang Q., Zhang Y. (2002). Properties of Raman spectra and laser-induced birefringence in polymethyl methacrylate optical fibres. J. Opt. A Pure Appl. Opt..

[62] Zhang Z., Shen W., Ye C., Luo Y., Li S., Li M., Xu C., Song Y. (2012). Large-area, crack-free polysilazane-based photonic crystals. J. Mater. Chem..

[63] Zhu Z., Zhang J., Wang C.F., Chen S. (2017). Construction of Hydrogen-Bond-Assisted Crack-Free Photonic Crystal Films and Their Performance on Fluorescence Enhancement Effect. Macromol. Mater. Eng..

[64] Hiltner P.A., Krieger I.M. (1969). Diffraction of light by ordered suspensions. J. Phy. Chem..

[65] Wang M., Meng F., Wu H., Wang J. (2016). Photonic Crystals with an Eye Pattern Similar to Peacock Tail Feathers. Crystals.

[66] Lee G.H., Choi T.M., Kim B., Han S.H., Lee J.M., Kim S.H. (2017). Chameleon-inspired mechanochromic photonic films composed of non-close-packed colloidal arrays. ACS Nano.

[67] Pointer M.R. (1981). A comparison of the CIE 1976 colour spaces. Color. Res. Appl..

